# Growth and differentiation of pluripotent embryonal carcinoma cells in the Snell dwarf mouse.

**DOI:** 10.1038/bjc.1984.204

**Published:** 1984-10

**Authors:** A. W. van der Kamp, S. C. van Buul-Offers, E. J. Roza-de Jongh, M. Feijlbrief, J. Branger, E. van Rongen

## Abstract

**Images:**


Br. J. Cancer (1984), **50**, 479–482

### Growth and differentiation of pluripotent embryonal carcinoma cells in the Snell dwarf mouse

A.W.M. van der Kamp[1], S.C. van Buul-Offers[2], E.J.M. Roza-de Jongh[1], M. Feijlbrief[2], J. Branger[2] & E. van Rongen[1]

[1]*Department of Cell Biology and Genetics, Erasmus University Rotterdam,* [2]*Department of Pediatrics, Wilhelmina Kinderziekenhuis, University of Utrecht, Utrecht, The Netherlands.*

**Summary** To investigate the influence of hormones on the process of cellular differentiation the growth and differentiation of a transplantable tumour, induced by inoculation of pluripotent mouse embryonal carcinoma (EC) cells have been studied in athymic nude mice and, normal and hypopituitary Snell dwarf mice. All athymic nude mice developed tumours independent of the numbers of cells inoculated. In contrast, the tumour percentage in normal Snell mice was lower, showing a dose-dependent increase of takes. In dwarfs tumour percentage was comparable with that observed in normal Snell mice.

The morphological differentiation of teratocarcinomas grown in athymic nude mice, normal and dwarfed Snell mice shows derivatives of all three germ layers next to undifferentiated embryonal carcinoma cells. This suggests that the pituitary hormonal deficiencies of the dwarfs (growth hormone, thyroid stimulating hormone and prolactin) did not influence the tumour induction nor the development of the different tissues present in this type of tumour.

The influence of the pituitary on induction and growth of several neoplasms has been studied in hypophysectomized rats and mice, and in hypopituitary Snell dwarf mice (Ball & Samuels, 1936; Korteweg & Thomas, 1939; Piantanelli & Fabris, 1978). In Snell dwarf mice (Snell, 1929) a hormonal influence on tumourigenesis is suggested, since the spontaneous tumour incidence in dwarfs was low compared to normals. For the induction of tumours by chemical carcinogens differences in incidence between dwarfs and normals became apparent in some cases depending on the type of carcinogen used and the route of application. With regard to transplantable tumours in dwarfs, growth and the number of takes of Ehrlich ascites tumours and sarcomas 180 was comparable to that found in normals (Turolla, 1960).

In this paper we report on the growth of a transplantable tumour, induced by inoculation of pluripotent mouse embryonal carcinoma cells in Snell dwarf and control mice. EC cells are pluripotent cells as demonstrated by the formation of a teratocarcinoma after subcutaneous injection of a single EC cell and by the formation of chimaeric mice after the injection of EC cells into mouse blastocysts (Kleinsmith & Pierce, 1964; Brinster, 1974). The homology between EC cells and embryonic cells is further supported by similarities in immunological, biochemical, ultrastructural and developmental characteristics (Artzt *et al.,* 1973; Graham, 1977; Evans & Kaufman, 1981). These developmental characteristics provide the possibility of studying the influence of hormones on the process of cellular differentiation within such a tumour.

Correspondence: A.W.M. van der Kamp, Department of Cell Biology and Genetics, Erasmus University, Dr Molewaterplein 50, P.O. Box 1738, 3000 DR Rotterdam, The Netherlands.
Received 21 February 1984; accepted 25 June 1984.

### Materials and methods

### *Experimental animals*

Parent mice, heterozygous for the dwarf gene (dw/+), with unknown genetical background, were a kind gift of Prof. Tanner (Institute of Child Health, London) in 1973. Two couples were inbred over 11 years in our department and before that for several years in Prof. Tanner's department. Therefore it is very likely but not absolutely certain that the dwarf gene will be the only difference between Snell normals and dwarfs. In this stock immunological T-cell deficiencies are absent (Schneider, 1976).

Snell normal mice and dwarfs (males and females) were bred and kept under standardized laboratory conditions (Buul-Offers, 1983). The Snell normal mice used are either homozygous normal (+/+) or heterozygous for the dwarf gene (dw/+), because there are no phenotypic traits available for the recognition of both types of mice. Athymic nude mice (C57BL/10LP background) were obtained from TNO, Zeist, The Netherlands.

© The Macmillan Press Ltd., 1984

*Cells and culture conditions*

PSMB is a pluripotent cell line cloned from tumour number OTT5568 (129/Sv mouse strain) (Stevens, 1970). The PSMB cell line was cultured on a feeder layer of STO cells. STO cells are thioguanine- and ouabain-resistant fibroblasts derived from a SIM mouse, isolated by Dr A. Bernstein (Ontario Cancer Institute). STO feeders were prepared by irradiation (Rö) of the cells with 30 Gy and seeded at $\sim 3.5 \times 10^6$ cells per 75 $cm^3$. PSMB cells ($5 \times 10^6$) were seeded onto a STO-feeder and passaged every third day. All cells were grown in Dulbecco's minimal essential medium (bicarbonate buffer, glucose 4.5 $g l^{-1}$) supplemented with 10% heat inactivated foetal calf serum (FCS), $10^{-4}$ M $\beta$-mercaptoethanol and antibiotics (100 IU of penicillin and 0.1 mg of streptomycin $ml^{-1}$ medium). The cells were routinely cultured in silicon rubber stoppered glass bottles. Cell cultures were split every two to three days by treatment for 5 min with $Ca^{2+}$ and $Mg^{2+}$ free PBS containing 1 mM EDTA and 0.25% trypsin. Cell lines were checked for mycoplasma by Hoechst staining (Chen, 1977).

*Tumour growth and differentiation*

Different numbers of PSMB cells ($1.10^6$–$5.10^6$) were inoculated s.c. in the neck of the experimental animals (age 9–11 weeks). Before inoculation the STO feeder cells were eliminated by passaging the PSMB cells once without feeder cells. The mice were inspected daily by palpation for the growth of tumours which were allowed to develop for periods from 7–100 days. The animals were killed under ether anaesthesia and the tumours excised. After fixation in 4% neutral buffered formaldehyde, tissue was embedded in paraffin and sections (5 $\mu$m) were stained with either H and E, Azan or alcian blue.

**Table I** Tumour takes of PSMB cells inoculated in nude mice, normal Snell and dwarf mice

| No. of cells inoculated | *No. of tumours/No. of mice* N | S | D |
|---|---|---|---|
| $1.10^6$ | 10/10 | 2/10 | 0/10 |
| $3.10^6$ | 10/10 | 10/19 | |
| $5.10^6$ | 4/4 | 2/3 | 8/10 |

N: nude mouse; S: normal Snell mouse; D: Snell dwarf mice.

Animals were scored as negative when tumour development did not occur within 3 months of inoculation.

**Table II** Tissue formation in teratocarcinomas in nude mice, normal Snell and Snell dwarf mice

| No. of cells inoculated | | *Tissue types* Neuronal | Epithelial | Cartilage | Muscle |
|---|---|---|---|---|---|
| $1 \times 10^6$ | N | ++ | ++ | + | ± |
| | S | + | + | + | + |
| | D | + | ++ | ++ | ++ |
| $3 \times 10^6$ | N | + | ++ | ++ | + |
| | S | + | ++ | + | + |
| | D | ++ | ++ | + | + |
| $5 \times 10^6$ | N | ++ | ++ | + | ± |
| | S | ++ | ++ | + | − |
| | D | ++ | + | + | ± |

N: nude mouse.
S: normal Snell mouse.
D: Snell dwarf mouse.
+: indicates the quantity of tissue.

### Results

In general tumours became detectable within 10 days after inoculation. Table I shows the number of takes; if animals did not develop a tumour within 3 months of inoculation they were scored as negative. The data show that all athymic nude mice develop tumours independent of the number of cells inoculated. In contrast, tumour percentage in normal Snell mice is lower, showing a dose-dependent increase of takes. In dwarfs the tumour percentage was comparable with that observed in normal Snell mice. Table II lists the tissue differentiations by PSMB cells in Snell normal, Snell dwarf and athymic nude mice. The morphological differentiation of teratocarcinomas grown in athymic nude mice shows derivatives of all three germ layers next to undifferentiated embryonal carcinoma cells. Tissue formation does occur in Snell normal and dwarf mice. In both mouse strains the formation of derivatives from all three germ layers is allowed without preference for, or selection against, any of the tissues observed on the athymic nude mice (Table II). Figure 1 shows some of the tissues formed in Snell dwarf mice.

### Discussion

Teratocarcinomas are composed of different types of tissue. such as nerve tissue, muscle, epithelium and cartilage. Growth and differentiation of normal cartilage is controlled by a variety of hormones and metabolic factors amongst which are growth

**Figure 1** Morphological differentiation of a teratocarcinoma in a Snell dwarf mouse. Number of PSMB cells inoculated $3 \times 10^6$. Left; neuronal tissue ( × 630), middle; cartilage ( × 400), right; ciliated epithelium ( × 1000).

hormone (GH)-dependent serum factors. These factors, called somatomedins, are mitogens which also stimulate amino acid transport and RNA and protein synthesis in different tissues (Salmon & Daughaday, 1957; Salmon & Duval, 1970; Buul-Offers, 1983).

It has been shown recently that amino acid transport and macromolecular synthesis, associated with the *in vitro* growth of a rat chondrosarcoma, were dependent on growth hormone-dependent serum factors as well as insulin (McClumbee & Lebovitz, 1980). Whether or not a similar hormonal dependency exists for the *in vivo* growth of this tumour is unknown.

In our *in vivo* system we found a similar percentage of tumour takes 3 months after inoculation in normal and dwarfed Snell mice, both of a lower level, however, compared to athymic nude mice. This suggests that the immunological systems of dwarf and normal Snell mice react in a similar way to counteract the development of this type of tumour. The large number of cells needed to generate tumours in these mice is probably necessary to overcome the immunological barrier due to difference in genetical background of the EC cells (129/Sv) and the Snell mice used. Undifferentiated mouse EC cells do not express H-2 antigens but do so upon differentiation (Jacob, 1977), therefore major genetic differences between the EC cells and the strains of mice used could have blocked the generation of differentiated tissues. Although EC cell transplantation within syngeneic strains of mice would have been preferable, the observed similarity in differentiation of teratocarcinomas in nude mice and dwarf and normal Snell mice indicates that no major immunological barrier exists. The similarity in morphological differentiation also suggests that the deficiency of dwarfs in growth hormone (GH) and consequently the somatomedians, thyroid stimulating hormone (TSH) and prolactin, did not influence the generation of the diversity of tissues present in this type of tumour.

Thanks are due to the Central Animal Center Rotterdam and to Mrs K. Linner for breeding the Snell mice; to Prof. Dr J.L. van den Brande for his stimulating criticism; to Ms R.J. Boucke for typing the manuscript and to Mr T. van Os for preparing the photographic illustrations.

### References

ARTZT, K., DUBOIS, P., BENNETT, D., CONDAMINE, H., BABINET, C. & JACOB, F. (1973). Surface antigens common to mouse cleavage embryos and primitive teratocarcinoma cells in culture. *Proc. Natl Acad. Sci.*, **70**, 2988.

BALL, H.W. & SAMUELS, L.T. (1936). The relation of the hypothesis to the growth of malignant tumours. III. The effect of hypophysectomy on autogenous tumours. *Am. J. Cancer*, **26**, 547.

BRINSTER, R.L. (1974). The effect of cells transferred into the mouse blastocyst on subsequent development. *J. Exp. Med.*, **140**, 1049.

BUUL-OFFERS, VAN, S. (1983). Hormonal and other inherited growth disturbances in mice, with special reference to the Snell dwarf mouse. A review. *Acta Endocrinol.*, (Suppl.), **258**, 1.

CHEN, T.R. (1977). In situ detection of mycoplasma contamination in cell cultures by fluorescent Hoechst 333258 stain. *Exp. Cell Res.*, **104**, 255.

EVANS, M.J. & KAUFMAN, M.H. (1981). Establishment in culture of pluripotential cells from mouse embryos. *Nature*, **292**, 154.

GRAHAM, C.F. (1977). Teratocarcinomas cells and normal mouse embryogenesis. In: *Concepts in Mammalian Embryogenesis*. (Ed. Sherman), Cambridge: MIT Press, p. 315.

JACOB, F. (1977). Mouse teratocarcinoma and embryonic antigens. *Immunol. Rev.*, **33**, 3.

KLEINSMITH, L.J. & PIERCE, G.B. (1964). Multipotentiality of single embryonal carcinoma cells. *Cancer Res.*, **24**, 1544.

KORTEWEG, R. & THOMAS, F. (1939). Tumor induction and tumor growth in hypophysectomized. *Am. J. Cancer*, **37**, 36.

McCLUMBEE, W.D. & LEBOVITZ, H.E. (1980). Hormone responsiveness of a transplantable rat chondrosarcoma. I. *In vitro* effects of growth hormone-dependent serum factors and insulin. *Endocrinology*, **106**, 905.

PIANTANELLI, L. & FABRIS, N. (1978). Hypopituitary dwarf and athymic mice and the study of the relationships among thymus, hormones and aging. *Birth Defects*, **14**, 315.

SALMON, W.D. & DAUGHADAY, W.H. (1957). A hormonally controlled serum factor which stimulates sulfate incorporation by cartilage *in vitro*. *J. Lab. Clin. Med.*, **49**, 825.

SALMON, W.D. & DUVAL, M.R. (1970). A serum fraction with "sulfation factor activity" stimulated *in vitro* incorporation of leucine and sulfate into protein-polysaccharide complexes, uridine into RNA and thymidine into DNA of costal cartilage from hypophysectomized rats. *Endocrinology*, **86**, 721.

SCHNEIDER, G.B. (1976). Immunological competence in Snell-Bagg pituitary dwarf mice: response to the contact sensitizing agent oxazolore. *Am. J. Anat.*, **145**, 371.

SNELL, G.D. (1929). Dwarf, a new mendelian recessive character of the house mouse. *Proc. Natl Acad. Sci.*, **15**, 733.

STEVENS, L.C. (1970). The development of transplantable teratocarcinomas from intratesticular grafts of pre- and postimplantation mouse embryos. *Develop. Biol.*, **21**, 364.

TUROLLA, E. (1960). Attecchimento e sviluppo del tumore ascito di Ehrlich e del sarcoma 180 in topi con nanismo ipofisario. *Tumori*, **46**, 20.


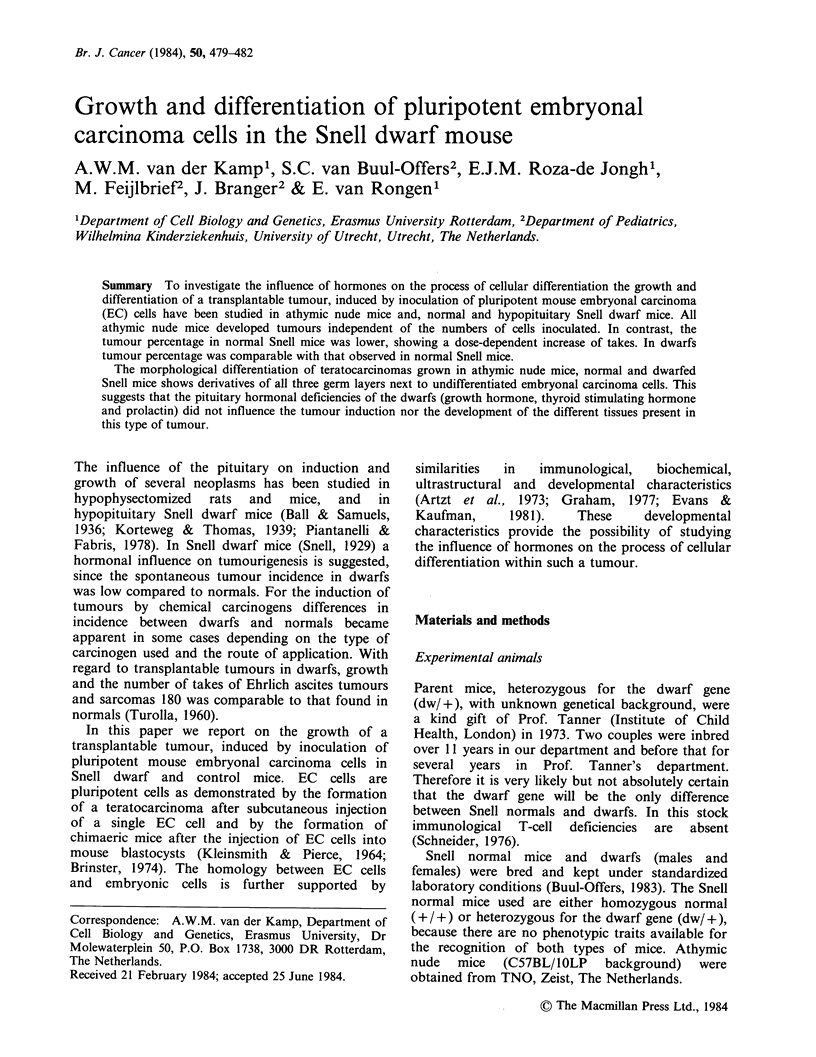

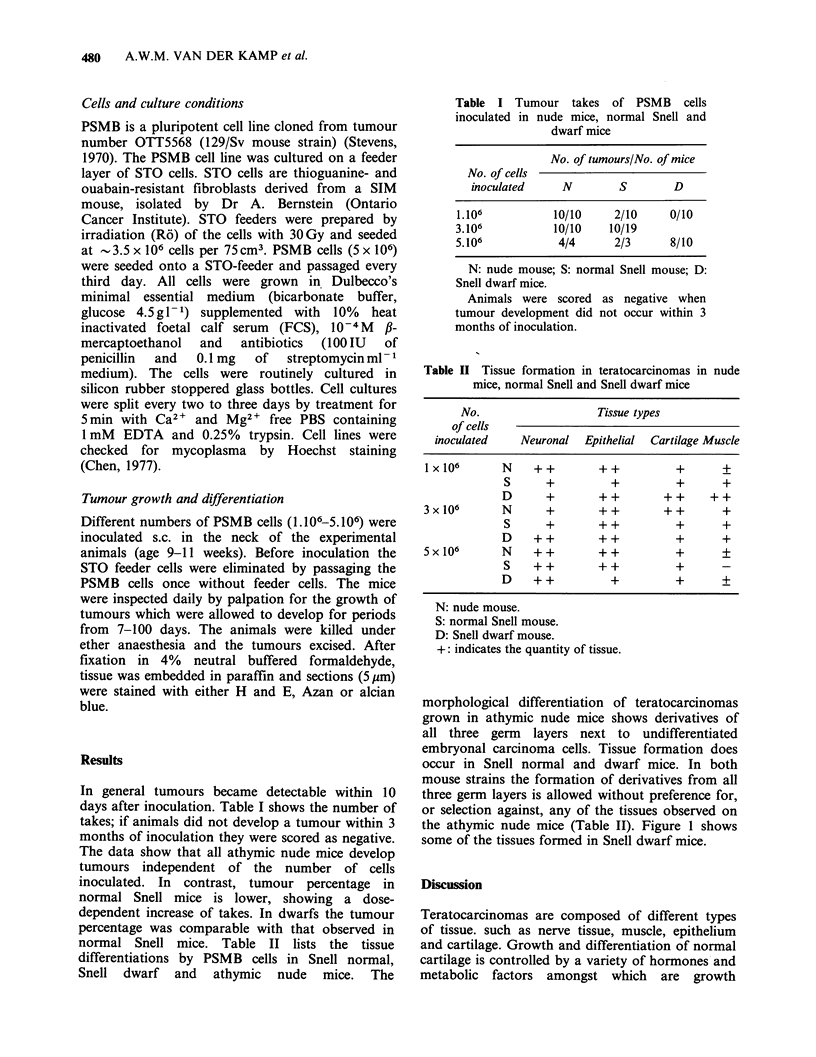

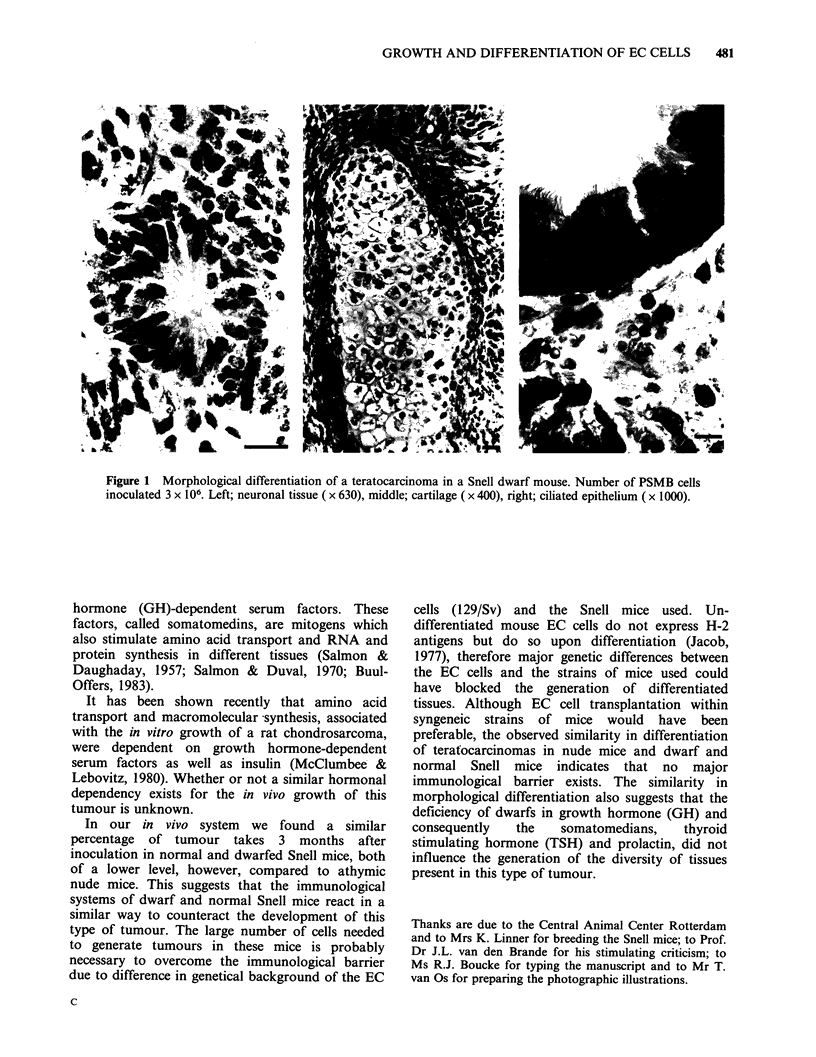

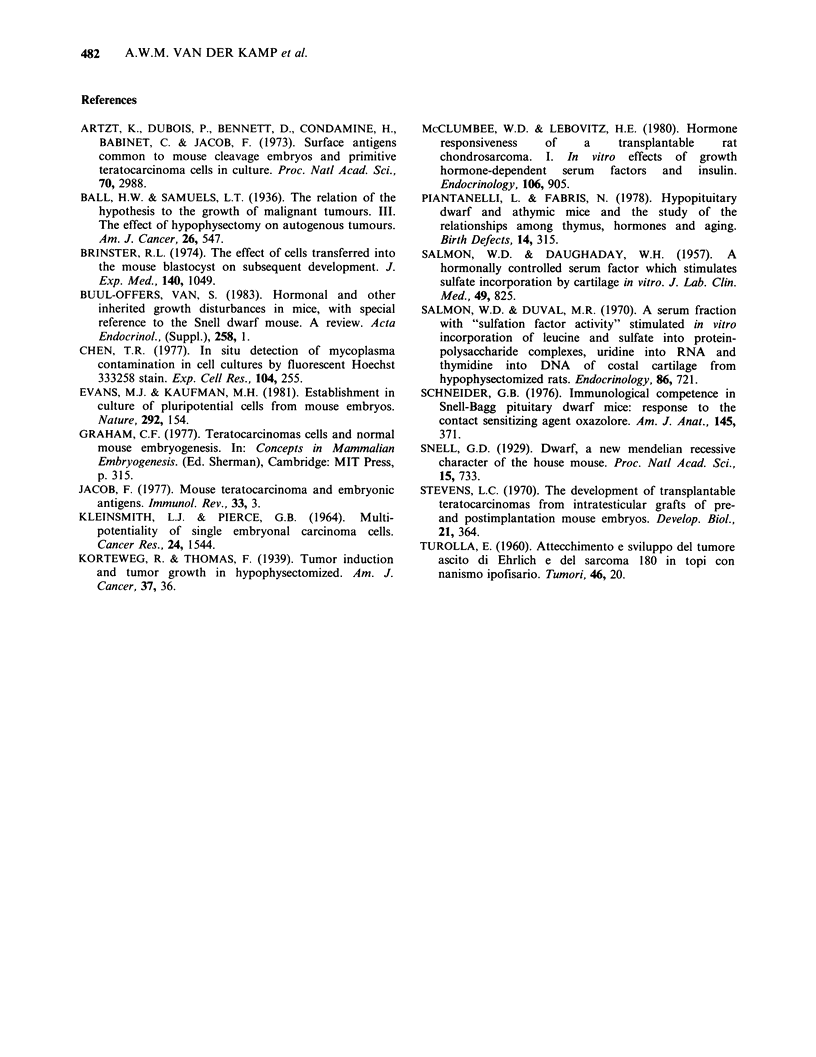


## References

[OCR_00290] Artzt K., Dubois P., Bennett D., Condamine H., Babinet C., Jacob F. (1973). Surface antigens common to mouse cleavage embryos and primitive teratocarcinoma cells in culture.. Proc Natl Acad Sci U S A.

[OCR_00303] Brinster R. L. (1974). The effect of cells transferred into the mouse blastocyst on subsequent development.. J Exp Med.

[OCR_00314] Chen T. R. (1977). In situ detection of mycoplasma contamination in cell cultures by fluorescent Hoechst 33258 stain.. Exp Cell Res.

[OCR_00319] Evans M. J., Kaufman M. H. (1981). Establishment in culture of pluripotential cells from mouse embryos.. Nature.

[OCR_00330] Jacob F. (1977). Mouse teratocarcinoma and embryonic antigens.. Immunol Rev.

[OCR_00334] KLEINSMITH L. J., PIERCE G. B. (1964). MULTIPOTENTIALITY OF SINGLE EMBRYONAL CARCINOMA CELLS.. Cancer Res.

[OCR_00344] McCumbee W. D., Lebovitz H. E. (1980). Hormone responsiveness of a transplantable rat chondrosarcoma. I. In vitro effects of growth hormone-dependent serum factors and insulin.. Endocrinology.

[OCR_00351] Piantanelli L., Fabris N. (1978). Hypopituitary dwarf and athymic nude mice and the study of the relationships among thymus, hormones, and aging.. Birth Defects Orig Artic Ser.

[OCR_00357] SALMON W. D., DAUGHADAY W. H. (1957). A hormonally controlled serum factor which stimulates sulfate incorporation by cartilage in vitro.. J Lab Clin Med.

[OCR_00363] Salmon W. D., DuVall M. R. (1970). A serum fraction with "sulfation factor activity" stimulates in vitro incorporation of leucine and sulfate into protein-polysaccharide complexes, uridine into RNA, and thymidine into DNA of costal cartilage from hypophysectomized rats.. Endocrinology.

[OCR_00371] Schneider G. B. (1976). Immunological competence in Snell-Bagg pituitary dwarf mice: response to the contact-sensitizing agent oxazolone.. Am J Anat.

[OCR_00377] Snell G. D. (1929). DWARF, A NEW MENDELIAN RECESSIVE CHARACTER OF THE HOUSE MOUSE.. Proc Natl Acad Sci U S A.

[OCR_00382] Stevens L. C. (1970). The development of transplantable teratocarcinomas from intratesticular grafts of pre- and postimplantation mouse embryos.. Dev Biol.

[OCR_00388] TUROLLA E. (1960). [Onset and development of Ehrlich ascites tumors and of sarcoma 180 in mice with pituitary nanism].. Tumori.

